# Supplementary material for: Bayesian machine learning analysis of single-molecule fluorescence colocalization images
Source: eLife. 2022 Mar 23;11:e73860. doi: 10.7554/eLife.73860 (PMC9183235; doi:10.7554/eLife.73860)
Supplement: Figure 7—source data 1. [file elife-73860-fig7-data1.pdf]

**input:** $D^{\text{raw}}$, $x^{\text{target,raw}}$, $y^{\text{target,raw}}$

**for all** frame$[F]$ **do**

  **for all** AOI$[N + N_{\text{c}}]$ **do**

    ▷ $(P - 1)/2$ is an AOI image center

    ▷ round function rounds to the closest integer

    $\text{shiftX} = \text{round}\left(x^{\text{target,raw}} - (P - 1)/2\right)$

    $\text{shiftY} = \text{round}\left(y^{\text{target,raw}} - (P - 1)/2\right)$

    $x^{\text{target}} = x^{\text{target,raw}} - \text{shiftX}$

    $y^{\text{target}} = y^{\text{target,raw}} - \text{shiftY}$

    $D_{\text{AOI}(n),\text{pixelX}(i),\text{pixelY}(j)} = D^{\text{raw}}_{\text{pixelX}(i+\text{shiftX}_{\text{AOI}(n)}),\text{pixelY}(j+\text{shiftY}_{\text{AOI}(n)})}$

**return** $D$, $x^{\text{target}}$, $y^{\text{target}}$
