# Supplementary material for: Bayesian machine learning analysis of single-molecule fluorescence colocalization images
Source: eLife. 2022 Mar 23;11:e73860. doi: 10.7554/eLife.73860 (PMC9183235; doi:10.7554/eLife.73860)
Supplement: Figure 8—source data 1. [file elife-73860-fig8-data1.pdf]

$g \sim \textbf{HalfNormal}(50)$ ▷ camera gain

$\sigma^{xy} \sim \textbf{Exponential}(1)$ ▷ standard deviation of on-target spot position (pixels)

$\pi \sim \textbf{Beta}(1/2, 1/2)$ ▷ mean specific binding probability

$\lambda \sim \textbf{Exponential}(1)$ ▷ non-specific binding density

for all AOI$[N + N_\mathsf{c}]$ **do**

$\quad \mu^b \sim \textbf{HalfNormal}(1000)$ ▷ mean background intensity

$\quad \sigma^b \sim \textbf{HalfNormal}(100)$ ▷ standard deviation of background intensity

$\quad$ **for all** frame$[F]$ **do**

$\quad\quad b \sim \textbf{Gamma}(\mu^b, \sigma^b)$ ▷ background intensity

$$z \sim \begin{cases} \textbf{Bernoulli}(\pi) & \text{on-target AOI} \\ \textbf{Bernoulli}(0) & \text{control off-target AOI} \end{cases}$$ ▷ target-specific spot presence

$$\theta \sim \begin{cases} \textbf{Categorical}\left(\left[1, 0, \dots, 0\right]\right) & z = 0 \\ \textbf{Categorical}\left(\left[0, \frac{1}{K}, \dots, \frac{1}{K}\right]\right) & z = 1 \end{cases}$$ ▷ target-specific spot index

$\quad\quad$ **for all** spot$[K]$ **do**

$$m_{\mathsf{spot}(k)} \sim \begin{cases} \textbf{Bernoulli}(1) & \theta = k \\ \textbf{Bernoulli}\left(\sum_{l=1}^{K} \dfrac{l \cdot \textbf{TruncPoisson}(l; \lambda, K)}{K}\right) & \theta = 0 \\ \textbf{Bernoulli}\left(\sum_{l=1}^{K-1} \dfrac{l \cdot \textbf{TruncPoisson}(l; \lambda, K-1)}{K-1}\right) & \text{otherwise} \end{cases}$$ ▷ spot presence

$\quad\quad\quad h \sim \textbf{HalfNormal}(10000)$ ▷ spot intensity

$\quad\quad\quad w \sim \textbf{Uniform}(0.75, 2.25)$ ▷ spot width

$$x_{\mathsf{spot}(k)} \sim \begin{cases} \textbf{AffineBeta}\left(0, \sigma^{xy}, -\dfrac{P+1}{2}, \dfrac{P+1}{2}\right) & \theta = k \\ \textbf{Uniform}\left(-\dfrac{P+1}{2}, \dfrac{P+1}{2}\right) & \theta \neq k \end{cases}$$ ▷ x-axis center

$$y_{\mathsf{spot}(k)} \sim \begin{cases} \textbf{AffineBeta}\left(0, \sigma^{xy}, -\dfrac{P+1}{2}, \dfrac{P+1}{2}\right) & \theta = k \\ \textbf{Uniform}\left(-\dfrac{P+1}{2}, \dfrac{P+1}{2}\right) & \theta \neq k \end{cases}$$ ▷ y-axis center

$\quad\quad\quad$ **for all** pixelX$[P]$ × pixelY$[P]$ **do**

$$\mu^S_{\mathsf{pixelX}(i),\mathsf{pixelY}(j)} = \frac{m \cdot h}{2\pi w^2} \exp\left(-\frac{(i - x - x^{\mathsf{target}})^2 + (j - y - y^{\mathsf{target}})^2}{2w^2}\right)$$ ▷ 2-D Gaussian spot

$\quad\quad$ **for all** pixelX$[P]$ × pixelY$[P]$ **do**

$\quad\quad\quad \delta \sim \textbf{Empirical}(\delta_{\mathsf{samples}}, \delta_{\mathsf{weights}})$ ▷ offset signal

$\quad\quad\quad \mu^I = b + \sum_{\mathsf{spot}} \mu^S$ ▷ mean pixel intensity w/o offset

$\quad\quad\quad I \sim \textbf{Gamma}(\mu^I, \sqrt{\mu^I \cdot g})$ ▷ pixel intensity w/o offset

$\quad\quad\quad D = \delta + I$ ▷ observed pixel intensity
