# Supplementary material for: Bayesian machine learning analysis of single-molecule fluorescence colocalization images
Source: eLife. 2022 Mar 23;11:e73860. doi: 10.7554/eLife.73860 (PMC9183235; doi:10.7554/eLife.73860)
Supplement: Figure 9—source data 1. [file elife-73860-fig9-data1.pdf]

Variational parameter initializations {initial value, constraint}:

$g_{\text{mean}} \leftarrow \{5, \quad \mathbb{R}_{>0}\}$; $g_{\text{beta}} \leftarrow \{100, \quad \mathbb{R}_{>0}\}$

$\sigma^{xy}_{\text{mean}} \leftarrow \{0, \quad (0, (P+1)/\sqrt{12})\}$; $\sigma^{xy}_{\text{beta}} \leftarrow \{100, \quad \mathbb{R}_{>2}\}$

$\pi_{\text{mean}} \leftarrow \{0.5, \quad [0,1]\}$; $\pi_{\text{size}} \leftarrow \{2, \quad \mathbb{R}_{>2}\}$

$\lambda_{\text{mean}} \leftarrow \{0.5, \quad \mathbb{R}_{>0}\}$; $\lambda_{\text{beta}} \leftarrow \{100, \quad \mathbb{R}_{>0}\}$

$\mu^b_{\text{mean}} \leftarrow \{\text{mean}(D)^{\text{AOI}[N]}, \quad \mathbb{R}_{>0}\}$; $\sigma^b_{\text{mean}} \leftarrow \{1^{\text{AOI}[N]}, \quad \mathbb{R}_{>0}\}$

$b_{\text{mean}} \leftarrow \{\text{mean}(D)^{\text{AOI}[N] \times \text{frame}[F]}, \quad \mathbb{R}_{>0}\}$

$b_{\text{beta}} \leftarrow \{1^{\text{AOI}[N] \times \text{frame}[F]}, \quad \mathbb{R}_{>0}\}$

$m_{\text{prob}} \leftarrow \{0.5^{\text{spot}[K] \times \text{AOI}[N] \times \text{frame}[F]}, \quad [0,1]\}$

$h_{\text{mean}} \leftarrow \{2000^{\text{spot}[K] \times \text{AOI}[N] \times \text{frame}[F]}, \quad \mathbb{R}_{>0}\}$

$h_{\text{beta}} \leftarrow \{0.001^{\text{spot}[K] \times \text{AOI}[N] \times \text{frame}[F]}, \quad \mathbb{R}_{>0}\}$

$w_{\text{mean}} \leftarrow \{1.5^{\text{spot}[K] \times \text{AOI}[N] \times \text{frame}[F]}, \quad [0.75, 2.25]\}$

$w_{\text{size}} \leftarrow \{100^{\text{spot}[K] \times \text{AOI}[N] \times \text{frame}[F]}, \quad \mathbb{R}_{>2}\}$

$x_{\text{mean}} \leftarrow \{0^{\text{spot}[K] \times \text{AOI}[N] \times \text{frame}[F]}, \quad [-(P+1)/2, (P+1)/2]\}$

$y_{\text{mean}} \leftarrow \{0^{\text{spot}[K] \times \text{AOI}[N] \times \text{frame}[F]}, \quad [-(P+1)/2, (P+1)/2]\}$

$xy_{\text{size}} \leftarrow \{200^{\text{spot}[K] \times \text{AOI}[N] \times \text{frame}[F]}, \quad \mathbb{R}_{>2}\}$

$g \sim \mathbf{Gamma}(g_{\text{mean}}, \sqrt{g_{\text{mean}}/g_{\text{beta}}})$ ▷ camera gain

$\sigma^{xy} \sim \mathbf{AffineBeta}(\sigma^{xy}_{\text{mean}}, \sigma^{xy}_{\text{size}}, 0, (P+1)/\sqrt{12})$ ▷ std of on-target spot position (pixels)

$\pi \sim \mathbf{Beta}(\pi_{\text{mean}}, \pi_{\text{size}})$ ▷ average specific binding probability

$\lambda \sim \mathbf{Gamma}(\lambda_{\text{mean}}, \sqrt{\lambda_{\text{mean}}/\lambda_{\text{beta}}})$ ▷ non-specific binding density

for all $\text{AOI}[N + N_{\text{c}}]$ **do**

  $\mu^b \sim \mathbf{Delta}(\mu^b_{\text{mean}})$ ▷ mean background intensity

  $\sigma^b \sim \mathbf{Delta}(\sigma^b_{\text{mean}})$ ▷ std of background intensity

  for all $\text{frame}[F]$ **do**

    $b \sim \mathbf{Gamma}(b_{\text{mean}}, \sqrt{b_{\text{mean}}/b_{\text{beta}}})$ ▷ background intensity

    for all $\text{spot}[K]$ **do**

      $m \sim \mathbf{Bernoulli}(m_{\text{prob}})$ ▷ spot presence

      if $m = 1$ **then**

        $h \sim \mathbf{Gamma}(h_{\text{mean}}, \sqrt{h_{\text{mean}}/h_{\text{beta}}})$ ▷ spot intensity

        $w \sim \mathbf{AffineBeta}(w_{\text{mean}}, w_{\text{size}}, 0.75, 2.25)$ ▷ spot width

        $x \sim \mathbf{AffineBeta}\left(x_{\text{mean}}, xy_{\text{size}}, -(P+1)/2, (P+1)/2\right)$ ▷ x-axis center

        $y \sim \mathbf{AffineBeta}\left(y_{\text{mean}}, xy_{\text{size}}, -(P+1)/2, (P+1)/2\right)$ ▷ y-axis center

      else if $m = 0$ **then**

        $h \sim \mathbf{HalfNormal}(10000)$

        $w \sim \mathbf{Uniform}(0.75, 2.25)$

        $x \sim \mathbf{Uniform}(-(P+1)/2, (P+1)/2)$

        $y \sim \mathbf{Uniform}(-(P+1)/2, (P+1)/2)$
